# A Parental-Report Questionnaire for Language Abilities and Pragmatics in Children and Adolescents with Autism Spectrum Disorders

**DOI:** 10.3390/brainsci13020196

**Published:** 2023-01-24

**Authors:** Aimee O’Shea, Claudia H. B. Holmes, Paul E. Engelhardt

**Affiliations:** 1 School of Psychology, University of East Anglia, Norwich NR4 7TJ, UK; 2 Department of Psychology and Language Sciences, University College London, London WC1E 6BT, UK

**Keywords:** Autism Spectrum Disorders, questionnaire validation, language ability, pragmatics, verbal children, social communication

## Abstract

The aim of this study was to test and validate a parental-report questionnaire, which assesses language abilities and pragmatics, in children with Autism Spectrum Disorders (ASD). We report two experiments: The first served as the initial test and the second sought to provide the first assessment of convergent validity. In total, we recruited 230 parents, where approximately two-thirds had a child with ASD. Results of factor analyses showed a consistent factor structure within each subscale, and the internal consistency was excellent for both sub-scales (Cronbach’s alpha >0.90). Convergent validity was assessed by correlating the results of the questionnaire with two sub-scales of the Autism Quotient questionnaire. The correlations were all greater than 0.60. The final version of the questionnaire (following exclusion of problematic items) contains 30 items (12 for language abilities and 18 for pragmatics). We conclude that the questionnaire is a concise and practical instrument for use in a variety of contexts for assessing language functioning and communication in children with ASD.

## 1. Introduction

Autism Spectrum Disorder (ASD) is characterised by restricted interests, repetitive patterns of behaviour, and persistent deficits in social communication and interaction across multiple contexts [1]. It is known that individuals with ASD show an atypical language profile. A universal hallmark of ASD is significant impairments in pragmatic aspects of language, including registering, turn-taking, and non-linguistic components, such as eye contact [2]. Functional language skills, on the other hand, are highly heterogenous across the spectrum. Many individuals with ASD develop average functional language skills in the expected trajectory of typically developing individuals, with no clinically significant problems in this area [3,4]. However, between 25% and 30% of children with ASD are either non-verbal or only minimally verbal [5]. Many children with ASD show language acquisition that is incongruent with typical developmental milestones, with the average age of first-word production being 38 months compared to 8–14 months in typically developing infants [6,7]. 

There is a strong body of evidence highlighting atypical structural language skills in children with ASD and across the lifespan with the use of phonology, morphemes, syntax, and semantics [4,8,9,10]. Significant impairments with these aspects of language functioning contribute to early identification and diagnosis of ASD, with the absence of words often being the primary concern for caregivers who suspect their child may have ASD [11]. Whilst the average age of diagnosis is 5 years old, a diagnosis can be obtained as early as 18–24 months, with social behaviours and language delay being indicative of atypical development in line with ASD symptomology [12,13,14].

There are currently no questionnaires for quick and reliable screening of language abilities and pragmatics in children. In this study, we developed an instrument to assess these things in children via parental report, and specifically, designed for use in children with ASD. The need for a reliable measure of language abilities and pragmatics to aid in early identification and diagnosis of ASD is clear. Impairments in language acquisition become increasingly problematic over time, and observed atypicalities in social communication, interaction, and behaviour should ideally result in immediate further evaluation [15,16]. Structural language impairments in typically developing children are associated with higher levels of anxiety, which progress into adolescence, and children with these impairments can be subject to peer rejection leading to fewer good quality relationships [17]. This may be exacerbated for individuals with ASD, who are already at a disproportionate risk of bullying, loneliness, and isolation, which can lead to serious mental health problems [18,19,20]. Pragmatic language skills have been shown to negatively predict anxiety levels and subsequent externalising behaviours in children with ASD, however there is evidence of a bi-directional relationship [21]. 

Educating caregivers on what verbal and non-verbal language cues to look for when identifying ASD symptoms in infants and children is crucial in predicting outcomes for affected children. This is because age of diagnosis is negatively related to outcomes [22]. In addition, when implemented early, interventions have been shown to be effective in improving language skills for children with ASD (for a review, see [23]). Encouraging parental involvement in this process is central to these more positive outcomes [24,25,26]. 

In this study, we investigate differences in language abilities and pragmatics between children with ASD and typically developing controls. Secondly, we investigated the psychometric properties (reliability and validity) of the instrument we developed, based on two empirical studies. Convergent validity was assessed, via correlations with Autism-Quotient (AQ) subscales of social skills and communication [27]. 

As mentioned previously, we believe that there are insufficient instruments designed for quick and efficient assessment of language abilities and pragmatics in ASD. We have identified three instruments that are frequently used for this purpose. The first is the Social Communication Questionnaire (SCQ) [28]. The SCQ is a screening instrument designed to be administered to caregivers to assess ASD symptomology in children. It requires a trained administrator, with guidelines suggesting the requirement of either a Bachelor’s degree, plus additional training, or a Master’s degree. The test kit is approximately $200 and scoring forms also require purchase. It has been criticised, however, for resulting in a high number of false-positives in clinical diagnoses [29,30]. The SCQ [28] does not clearly delineate language abilities and pragmatics into two separate domains. Language abilities and pragmatics are distinct and there are variable presentations of both across the spectrum, so research on language in ASD that does not make this distinction is hard to interpret and draw conclusions from. The second instrument is the Child Communication Checklist (CCC-2) [31]. The CCC-2 is a 70-item questionnaire designed to be administered by a caregiver to screen for communication disorders in children. Again, however, the test kit is expensive and requires a Bachelor’s or Master’s degree to access it, making this a potential barrier to diagnosis. A further instrument which is designed to assess ASD symptomology is the Broad Autism Phenotype Questionnaire (BAPQ) [32]. The Broad Autism Phenotype (BAP) is a set of subclinical autistic traits that reflect potential genetic liability to ASD. The BAPQ is designed to screen relatives of autistic individuals for the BAP across general domains of ASD symptoms: social behaviours, rigid behaviours and interests, and pragmatic communication. We believe that our questionnaire is unique in being a comprehensive focus on language abilities and pragmatics, which are key identifiable symptoms that lead to official diagnosis of ASD. Furthermore, the BAPQ is designed for use in adults, whereas we strongly believe that having the capacity to utilise such a screening instrument with children is necessary when early intervention is critical to outcomes [22]. We have identified other less-prominent instruments that may be used for this purpose. We have included a table which lists them (along with the SCC, BAPQ and CCC), their main properties, and their strengths and weaknesses for comparison in the Supplementary Materials, Section A.

Other tests for assessing language are the Clinical Evaluation of Language Fundamentals and the Test for Reception of Grammar (for a review, see [33]). However, again, these tests have substantial cost implications and require training to administer. Some studies have also collected spontaneous speech samples (e.g., [34]). The purpose of doing so is to understand whether the individual with ASD is able to communicate with intent, stay on topic, provide sufficient information, and understand humour and irony. Tests for assessing pragmatics include the Test of Pragmatic Language and Comprehensive Assessment of Spoken Language. These tests also have cost implications and require 45 min to 1 h to administer. In addition, these assessments can be challenging to administer to individuals with ASD. This is because it is often difficult for children with ASD to interact with an experimenter, and they may experience motivational and attentional difficulties [35]. Parent-report questionnaires, in contrast, are easy for the individual with ASD, as they allow for the observation of natural behaviour in environments that are familiar and comfortable.

In short, these standardised tests require substantial resources and effort to administer. The process of receiving an ASD diagnosis is often long and complex requiring costly expert clinical assessments. This is often a barrier to diagnosis for some individuals and their families [14], which is especially problematic when access to early interventions is crucial for outcomes. The capacity of diagnostic services has been stretched, particularly in and following the 2020–2021 COVID-19 pandemic. These issues have led to the need for screening measures designed to quickly and efficiently identify individuals with ASD symptoms, in order to guide referrals to clinical experts. The main goal of this study is not to replace existing measures, but instead, to provide a quick, easy, reliable, and cost-efficient way for researchers and clinicians to assess language abilities and pragmatics. 

Two experiments were conducted. Experiment 1 presents the results of the first study using the new questionnaire. The first version of the questionnaire had 34 items containing two sub-scales (language ability and pragmatics). We recruited parents who had either a typically developing child or a child with ASD. Experiment 1 provided the initial assessment of the questionnaire, in which we examined children with ASD in comparison to typically developing children, as well as the factor structure and internal reliability of each sub-scale. In the second experiment, we revised the questionnaire based on Experiment 1 results. In short, we removed two items and re-ran the questionnaire on a second sample in order to validate the questionnaire. The second experiment also assessed two sub-scales from the AQ, namely social skills and communication. Correlations between these sub-scales and the sub-scales of the language and pragmatics questionnaire provide the first validation of convergent validity. We expected pragmatics to be more impaired than language abilities, given that no individuals in the ASD sample(s) were non-verbal.

## 2. Experiment 1

The first study recruited parents in order to provide a first test of our questionnaire. Parents of children completed a few simple demographic questions, and then the language and pragmatics questionnaire. All did so online via Qualtrics. We expected to observe significant differences between children with ASD and typically developing children. This study provided the initial step in demonstrating that the questionnaire operated as intended. The second step in Experiment 1 examined the psychometric properties of the questionnaire. To do so, we conducted factor analyses to assess whether individual items on each sub-scale patterned together. Finally, we assessed the internal reliability. 

### 2.1. Methods

#### 2.1.1. Participants

A volunteer sample of 137 parents were recruited. Seventy-seven were the parent (or guardian) of a child with ASD, and 60 were the parent of a typically developing child. The recruitment of parents (or guardians), who had a child with ASD was performed via local ASD support groups. The group coordinators agreed to circulate the link to the questionnaire via email to their group members. In addition, several personal contacts, who had a child with ASD were also approached to take part by email. The recruitment of the parents/guardians, whose child did not have ASD, was performed via a convenience sample, which involved sending the questionnaire to contacts in a local elementary school (in the southeast region of England). The age and gender of the children are shown in Table 1. Further demographic information about the sample is presented in the Supplementary Materials, Section B. 

#### 2.1.2. Materials 

Language and Pragmatics Questionnaire. The questionnaire consisted of two parts: language ability (14 items) and pragmatics (20 items). The questionnaire was designed for parents to complete with respect to their child. In each sub-scale, a higher score indicates a lower ability of the child. A 4-point Likert scale ranging from “Strongly Agree” (coded as 4) to “Strongly Disagree” (coded as 1) was used to indicate how much parents agreed with each statement. Nine questions were reversed coded (8, 9, 13, 19, 21, 22, 28, 29, 30, 31). Item selection and initial content validation was performed by reviewing the diagnostic criteria for ASD and several empirical studies, which examined language impairments in ASD. 

#### 2.1.3. Design and Procedure

Before data collection, ethical approval was granted. Participants were approached by email, asking them whether they would like to take part in the study. Those who decided to take part were able to click on a link, which took them directly to the questionnaire. Before completing the questionnaire, participants were shown the information sheet, which provided an outline of the study, followed by the consent form. The consent form made it clear that participation was voluntary, and participants were free to withdraw at any time, without giving reason. It also informed them to leave blank any questions which they felt uncomfortable answering or were otherwise not applicable. The participants were asked to tick a box to confirm their participation in the study, and provide the last four letters of their surname, along with their date of birth. Following this, participants completed the questionnaire. In addition to the language and pragmatics questions, parents also completed another unrelated questionnaire. 

The participants were shown the debrief after completing the questionnaire. This explained what the study was investigating, and provided the researchers contact details, along with information about relevant support groups. The debrief page also offered more information about withdrawal. The last point of withdrawal was one month after participation. If the participants decided that they wanted to withdraw their data, then they could email the last four letters of their surname and their dates of birth to the researcher, so that their data could be removed from the study.

### 2.2. Results

#### 2.2.1. ASD vs. Control

**Figure 1:**
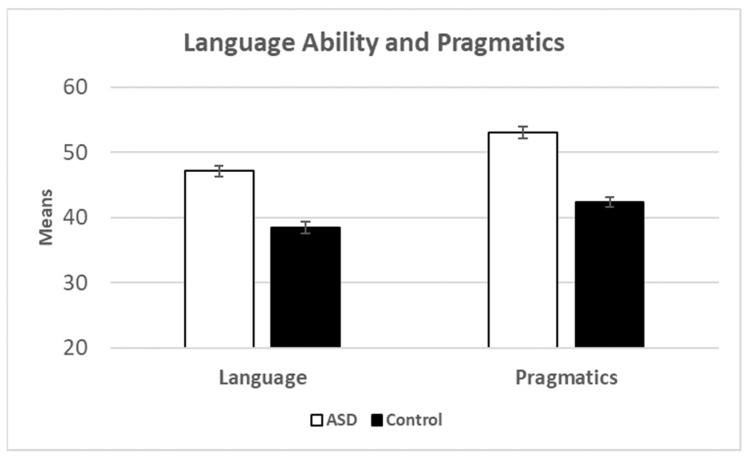
Means for language ability and pragmatics broken down by group. Error bars show the standard error of the mean.

**Figure 2:**
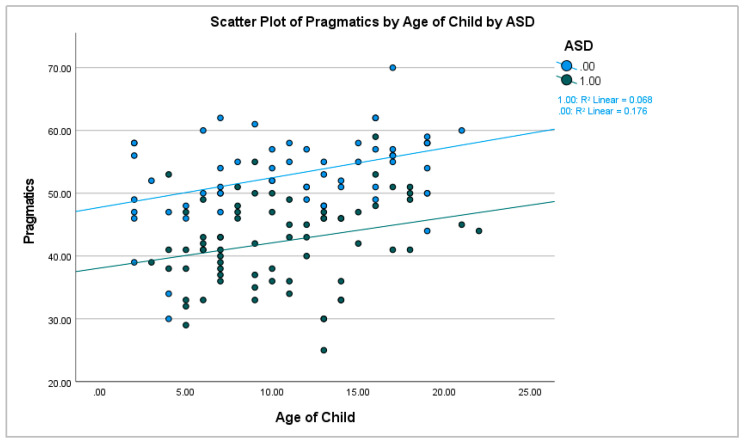
Scatterplot of the age by group effect on pragmatics.

We began the analysis by comparing children with ASD to controls. Before, proceeding with the inferential analyses, we checked the data for outliers using ± 3 SDs from the mean, we also ensured that the data were normally distributed. The means are presented in Figure 1. Independent sample *t*-tests showed significant differences: language ability *t*(131) = 6.87, *p* < 0.001 (*d* = 1.20) and pragmatics *t*(131) = 9.08, *p* < 0.001 (*d* = 1.58). As a follow up analysis, we conducted two ANCOVAs with the inclusion of age (of the child) and gender as a covariates. For language abilities, the ASD vs. control effect remained significant *F*(1,128) = 36.57, *p* < 0.001, *η^2^* = 0.22 but age *F*(1,128) = 3.39, *p* = 0.068, *η^2^* = 0.026 and gender *F*(1,128) = 3.11, *p* = 0.080, *η^2^* = 0.024 were not significant. For pragmatics, the ASD vs. control effect remained significant *F*(1,128) = 74.76, *p* < 0.001, *η^2^* = 0.37, and gender was not significant *F*(1,128) = 0.47, *p* = 0.495, *η^2^* = 0.004. However, age was a significant factor with respect to pragmatics *F*(1,128) = 13.46, *p* < 0.001, *η^2^* = 0.095 (see Figure 2). 

Discriminant Analysis. The two groups differed in language abilities and pragmatics. We conducted a discriminant analysis in order to determine whether the results from the questionnaire could be used to predict group classification (ASD vs. control). The results from that analysis showed that 79.5% of control cases were correctly predicted, and 86.7% of ASD cases were correctly predicted. Thus, in total, 82.7% (approximately 4 out of every 5 cases) were correctly predicted (Wilk’s λ = 0.592). 

#### 2.2.2. Psychometric Analysis

Factor Structure. We conducted two factor analyses (one for each sub-scale) to assess the underlying factor structure, and to validate the sub-scale items as loading on the same construct (i.e., language abilities and pragmatics, respectively). Results for the factor analysis on language ability items showed that one item (item 7) did not load significantly on Factor 1. The factor loading of item 7 was −0.151. We therefore removed that item. The results for the pragmatics sub-scale likewise showed one item (item 33) that did not load significantly on Factor 1. The factor loading of item 33 was 0.141. This item was also removed. The results of the two factor analyses (following item removal) are shown in Table 2 and Table 3. Both showed that there were three factors, which had Eigenvalues > 1. However, Factor 1 in each analysis accounted for approximately 50% of the variance, and the other two factors accounted for a comparably smaller amount of variance. 

For the interpretation of factor loadings, we used the recommendations of Stevens [36] for determining significance. We interpreted factor loadings of 0.434 and greater as significant, which we highlighted with grey shading in Table 3. As can be seen in Table 3, all items loaded highly and significantly on Factor 1 for both sub-scales. For language abilities, there were four significant factor loadings on Factor 2 and two significant factor loadings on Factor 3. However, for all five of these “additional” significant factor loadings, the factor loading was numerically lower than the factor loading on Factor 1. In addition, the factor loadings were a mix of positive and negative for Factor 2. Similar results obtained for the pragmatics sub-scale. Thus, this analysis shows that the items load primarily on the same construct (or factor) and show a high degree of overlapping variance. 

Internal Consistency. We examined the internal reliability of the items on each sub-scale. We found that Cronbach’s alpha for language ability was 0.90. Similarly, for pragmatics, Cronbach’s alpha was 0.94. This shows excellent internal reliability for both sub-scales. 

### 2.3. Discussion

The results of Experiment 1 revealed that the questionnaire showed significant differences between children with ASD and typically developing controls. We also observed that age had a significant effect on pragmatics sub-scale, but results for language ability showed that age and gender were not significant. Moreover, the discriminant analysis showed that approximately 4 out of 5 cases could be correctly predicted just based on the two sub-scales of the questionnaire. This indicates reasonably good diagnostic utility of the questionnaire. 

The psychometric analysis of the questionnaire showed that there were two items that did not load on the expected factors (see Supplementary Materials, Section C). Results of factor analyses on the remaining 32 items showed that in general Factor 1 (of each sub-scale) accounted for approximately 50% of the variance, and that the other two significant factors showed very few (and inconsistent) significant factor loadings. In short, Factor 1 was substantially larger than Factors 2 and 3, and all items loaded significantly on Factor 1. These results show that the sub-scale questions were highly related to one another and tap into the same underlying construct (i.e., language abilities and pragmatics, respectively). Internal consistency for the sub-scales was good, as the alphas were >0.90. We used the revised questionnaire in Experiment 2. 

## 3. Experiment 2

The purpose of the second study was to provide an initial validation of the questionnaire. We did so by testing a second sample of participants, and by simultaneously having parents complete two sub-scales of the AQ questionnaire [27]. The sub-scales were social skills and communication. The general procedure of Experiment 2 was similar to Experiment 1. To establish convergent validity, a new instrument should be compared to closely related constructs (i.e., a similar questionnaire). For Experiment 2, we examined convergent validity with the AQ.

### 3.1. Methods

#### 3.1.1. Participants

One hundred and twenty-eight parents, all of whom were parents to at least one child, were recruited via opportunity sampling. Exclusion criteria included answering less than half of the questions and answering *no* to the question “is your child verbal”. The data from 31 participants was removed because of these exclusions, and thus, the final sample consisted of 97 participants. The age and gender of the 97 children are presented in Table 4. Further demographic information about the sample is presented in the Supplementary Materials, Section D. 

#### 3.1.2. Materials

Language and Pragmatics Questionnaire. The questionnaire consisted of two parts: language ability (13 statements) and pragmatics (19 statements). 

Autism Quotient. The AQ is a self-report measure of ASD traits, consisting of 50 items assessing ASD symptomology in five areas: social skills, attention switching, attention to detail, communication, and imagination. Answers are given on a four-point Likert scale from “Definitely Agree” to “Definitely Disagree’. “Definitely Agree” and “Slightly Agree” score one point on certain items. “Definitely Disagree” and “Slightly Disagree” score one point on other items. Scores on the AQ are summed and can range from 0 to 50 with a higher score indicating that the individual possesses a higher level of ASD traits. For the purposes of the study, the two sub-scales of the AQ were also summed. 

#### 3.1.3. Design and Procedure 

A recruitment flyer for this study was shared by the researcher on Facebook and LinkedIn. Additionally, it was emailed to the headteachers of local primary schools and published in the closed Charity Facebook page “SPACE’, a support group for parents of children with ASD and other neurodivergent disorders in Hertfordshire. The link to the online study was attached to this flyer. Because this study was conducted at the height of the COVID pandemic, we had a number of participants who were currently awaiting an ASD diagnosis, and thus, were strongly suspected of having ASD. We included these “strongly suspected” cases of ASD in the study. 

Participants first read the information sheet, which explained that the aim of the study was to explore the predictors of language development in children with and without ASD and described the measures that would be used. It also explained that the study would take no longer than 20 min to complete and participants could withdraw at any point. Participants gave informed consent and confirmed that they were over the age of 18 before giving demographic information based on their child. If participants answered “no” to the question is your child verbal they were directed to the end of the study and were advised to email the researcher for the debrief. 

Upon completion of the demographic questionnaire, participants were presented with the social skills and communication items from the AQ (see Supplementary Materials, Section F), followed by the language abilities and pragmatics items. They were then presented with the debrief, which reiterated the aims of the study and the right to withdraw. Participants were asked one final time to consent to their data being used. 

### 3.2. Results

#### 3.2.1. ASD vs. Control

**Figure 3:**
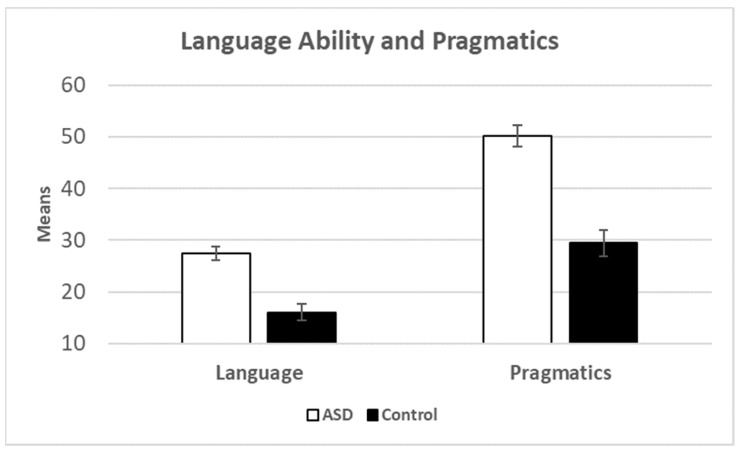
Means for language ability and pragmatics broken down by group. Error bars show the standard error of the mean.

We began the analysis by comparing the ASD participants to controls. The means for both sub-scales are presented in Figure 3. Independent sample *t*-tests showed significant differences: language ability *t*(95) = -5.50, *p* < 0.001 (*d* = -1.25) and pragmatics *t*(95) = −7.13, *p* < 0.001 (*d* = −1.62). As a second analysis, we conducted the same analysis but with the inclusion of gender as a covariate. (There were too many missing data points to include age in this analysis.) For language abilities, the ASD effect remained significant *F*(1.94) = 25.96, *p <* 0.001, *η^2^* = 0.22, but gender was not significant *F*(1.94) = 3.26, *p* = 0.074, *η^2^* = 0.034. For pragmatics, the ASD effect remained significant *F*(1.94) = 45.80, *p* < 0.001, *η^2^* = 0.33, and gender was again not significant *F*(1.94) = 1.39, *p* = 0.242, *η^2^* = 0.015. 

Discriminant Analysis. The two groups differed in language abilities and pragmatics. We conducted a discriminant analysis in order to determine whether the results from the questionnaire could be used to predict group classification (ASD vs. control). The results from that analysis showed that 88.9% of controls were correctly predicted, and 82.9% of ASD cases were correctly predicted from the two sub-scales. In total, 84.5% (approximately 4 out of every 5 cases) were correctly predicted (Wilk’s λ = 0.649).

#### 3.2.2. Psychometric Analysis

*Factor Structure.* We conducted two factor analyses (one for each sub-scale) to assess the underlying factor structure. The results of the factor analyses are shown in Table 5 and Table 6. For language abilities, there were two significant factors, and for pragmatics, there were three factors. However, Factor 1 in each analysis accounted for approximately 50% of the variance, and the other factors accounted for a much smaller proportion of variance. For the factor loadings, we interpreted factor loadings of 0.512 and greater as significant [34]. As can be seen in the Tables below, for language abilities, item 4 did not load significantly on Factor 1. Likewise, for pragmatics, items 16 and 22 did not load significantly on Factor 1. We return to these items in the General Discussion, but in the end, we elected to remove items 4 and 22 from the final version of the questionnaire (see Supplementary Materials, Section E). As in the previous study, there were several significant factor loadings on Factors 2 and 3. The significant ones for language abilities were item 2, 3, and 4. Items 2 and 3 showed negative factor loadings, and item 4 was the item with a non-significant loading on Factor 1. For pragmatics, item 16 showed a significant (negative) loading on Factor 2, item 22 showed a large and significant loading on Factor 3, and item 28 showed a nearly identical positive loading on Factor 2, as compared to Factor 1. Overall, virtually all items showed significant and high factor loadings on Factor 1.

Internal Consistency. We examined the internal reliability of the sub-scales. We found that Cronbach’s alpha for the language ability items was 0.90. Similarly, for the pragmatics items, Cronbach’s alpha was 0.93. These analyses show excellent internal reliability.

**Figure 4:**
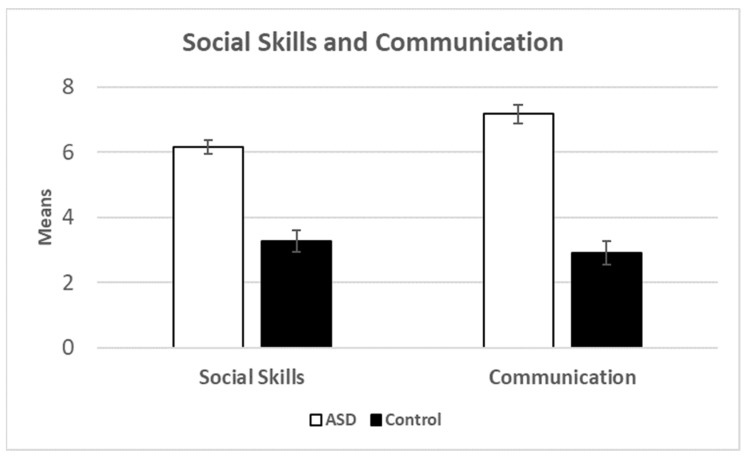
Means for social skills and communication broken down by group. Error bars show the standard error of the mean.

Concurrent Validity. We observed significant differences between children with ASD and controls for the two AQ sub-scales: social skills t(95) = -8.64, p < 0.001 (d = -1.96) and communication t(95) = -10.23, p < 0.001 (d = -2.31) (see Figure 4). Social skills correlated with language ability r = 0.60, p < 0.001 and pragmatics r = 0.69, p < 0.001. Communication correlated with language ability r = 0.65, p < 0.001 and pragmatics r = 0.74, p < 0.001. For both analyses, we expected pragmatics to correlate more highly with the AQ sub-scales as compared to language abilities, and that expectation was confirmed. Thus, the language and pragmatics questionnaire patterns similarly to the AQ, which given that is it designed to assess ASD symptomology, demonstrates good convergent validity. 

### 3.3. Discussion

The results of Experiment 2 were largely similar to those of Experiment 1. We observed significant differences between children with ASD and controls on both language abilities and pragmatics. Both showed nearly identical effect sizes, as compared to Experiment 1. Moreover, the discriminant analysis showed that 4 out of 5 cases could be correctly predicted based on the means of the questionnaire. 

The results of the factor analyses also showed similar results as Experiment 1. However, there were a couple of unexpected findings. In particular, two of the items (items 4 and 22) did not show significant factor loadings on “Factor 1”. These two items did show significant factor loadings in Experiment 1. Overall, however, the results of the factor analyses did show similar results in terms of the proportion of variance explained, and all of the other items did show significant factor loadings, as compared to the results of the factor analyses in Experiment 1. In terms of reliability, there was again excellent internal reliability for both sub-scales. 

The examination of convergent validity with the AQ questionnaire, showed that the language abilities and pragmatics were highly correlated with the social skills and communication sub-scales of the AQ. These correlations mirror the large effects sizes between groups on both the AQ and the language abilities and pragmatics sub-scales, and suggest that both questionnaires tap into similar underlying constructs (i.e., language and communication issues associated with ASD symptomology). In short, we feel that these results demonstrate good convergent validity of our questionnaire. 

## 4. General Discussion

The current study reported the initial development and validation of the language and pragmatics questionnaire, a new questionnaire that provides a quantitative measurement of individual differences in language abilities and pragmatics in children with ASD based on parental report. Language-related problems are common across the lifespan in individuals with ASD, and they are easy to screen for in childhood where certain developmental atypicalities, such as language delay, are manifestations of the later social communication difficulties, which are diagnostic characteristics of ASD. Atypical language abilities and pragmatics have long been identified in ASD as being problematic issues. Impairments in abilities within these domains have been related to poorer outcomes in language-specific developmental milestones, educational achievements, and social integration [6,11,17,37]. Importantly, however, these negative outcomes can be mitigated by protective factors, such as age of diagnosis, early intervention, and parental involvement [22,23,24]. Therefore, we believe that an instrument that assists in supporting protective factors, and that is specifically designed to quickly and reliably screen for these issues, will be beneficial in presenting better outcomes for individuals with ASD, ultimately leading to less debilitation in wider societal contexts. 

We investigated this new instrument as a predictor of ASD cases in two samples of children, approximately two-thirds of which had (or were strongly suspected to have) ASD. Our objective was to determine whether this new questionnaire would be an effective means of screening these two key issues in ASD. Across two samples, we observed significant differences between typically developing children and children with ASD. Moreover, the instrument was shown to have (1) excellent internal reliability (alpha’s being 0.90 or greater), (2) strong predictive validity, given that 4 out of 5 individuals could be correctly classified, and (3) good convergent validity, as assessed via correlations with the AQ questionnaire (correlations >0.60). We feel that the main benefit of this questionnaire is the quick and easy administration (i.e., parental report taking approximately 10–12 min), and the fact that it is free and does not require a trained researcher to administer. Based on these positive results, we strongly suspect that this questionnaire can be utilised as a screening device in routine clinical assessments, in educational contexts, and for research purposes, similar to the way in which the AQ is so often used in adult samples. 

Based on the results of two sets of factor analyses, we proposed to eliminate several questions from the final version of the questionnaire (see Supplementary Materials, Section C for further details). This leaves 30 questions in the final version (LAP 1.1.0), which is available in Section E of the Supplementary Materials. Moreover, the factor analyses showed largely consistent results, and that, outside of the four excluded items, all items loaded significantly on Factor 1, which accounted for approximately half of the variance. The one exception to this was item 16 in Experiment 2. We elected to retain this item in the questionnaire given (1) that it did load significantly in Experiment 1 and (2) that the factor loading just missed significance and did not show a significant positive loading on another factor. (Note also that with a larger sample size, the factor loading in Experiment 2 would have been significant.) The results of the factor analyses suggest that items within each sub-scale are doing a reasonably good job of assessing the intended constructs. We also argued that significant factor loading on Factors 2 and 3 did not elucidate any meaningful sub-categories of items. Perhaps, the only exception to this is the significant (positive) factor loadings on Factor 2 (Experiment 1). Item 8 (responsive to speech) and Item 13 (follow directions) would both seem to involve interaction with an interlocuter. We think that this possibility would need to be addressed in future studies, and these items did not pattern, as such, in Experiment 2. 

To this point in the discussion, we have focused on the positive results achieved in Experiments 1 and 2. We also think it is important to highlight the aspects of the study that do not completely fit with the overall narrative, and perhaps, lead to further questions and/or open potential issues with the data. The most obvious of these is the missing “age” data in Experiment 2. This was primarily due to the age question appearing at a place in the questionnaire where many parents, simply did not see it. Once the problem was brought to our attention, we changed the placement of this question and made it a required field. The missing age data prevented us from including it in the analyses of Experiment 2, which is unfortunate given there was a significant age effect on the pragmatics sub-scale in Experiment 1 (see Figure 2). With respect to gender, it did not produce a significant effect when included as a covariate in our models. However, it had a larger effect on language abilities, compared to pragmatics, in both experiments. It is important to note, that the gender effect was inconsistent. In Experiment 1, females tended to show slightly worse language abilities, and in Experiment 2, the reverse occurred. However, gender was not statistically significant, and the mean differences were comparatively less than the between groups analysis. Finally, we think it is important to consider that our ASD samples contained a surprisingly high number of females, which is quite unusual. In fact, our ASD samples had a more even gender split, as compared to our control samples. This is quite unusual for an ASD study.

### 4.1. Questionnaire Interpretation

There are a couple of key findings, which need to be addressed with respect to how the questionnaire results for a particular individual can be interpreted. The first is that there were relatively large differences in the means between Experiment 1 and Experiment 2. Essentially, the means for Experiment 1 were higher than those in Experiment 2. We do not think that these differences are a problem because of a couple of key points with respect to the samples. Most importantly, the summed results for Experiment 2 were based on two fewer items, and hence, means were expected to be lower. Second, the controls for Experiment 2 were substantially younger, and across all of our data, we observed that parents generally rated the language abilities and pragmatics of older children as being worse (more atypical), even for controls, as compared to younger children. At first glance, this is a counterintuitive result given that these abilities should improve across the course of development (i.e., become more normalised over time). This is most easily seen in Figure 2, where the slopes of the best fit lines are positive. In early language development there is inherently more variability between children, and over the course of development, children become more adultlike. In general, much of language development is completed by the age of 8. Parents, then, likely become more aware of their child’s issues as they get older because (1) they have more interactions with their children and those interactions become more adultlike, and (2) they likely have more insights into how their child may or may not differ from other children of the same age. In short, in early childhood parents generally feel better about their child’s language and pragmatic abilities, and as time progresses, they can more easily identify, and cast a critical eye, toward deficiencies or issues. Thus, positively sloped lines are likely not as problematic as they would seem at first sight. 

**Figure 5:**
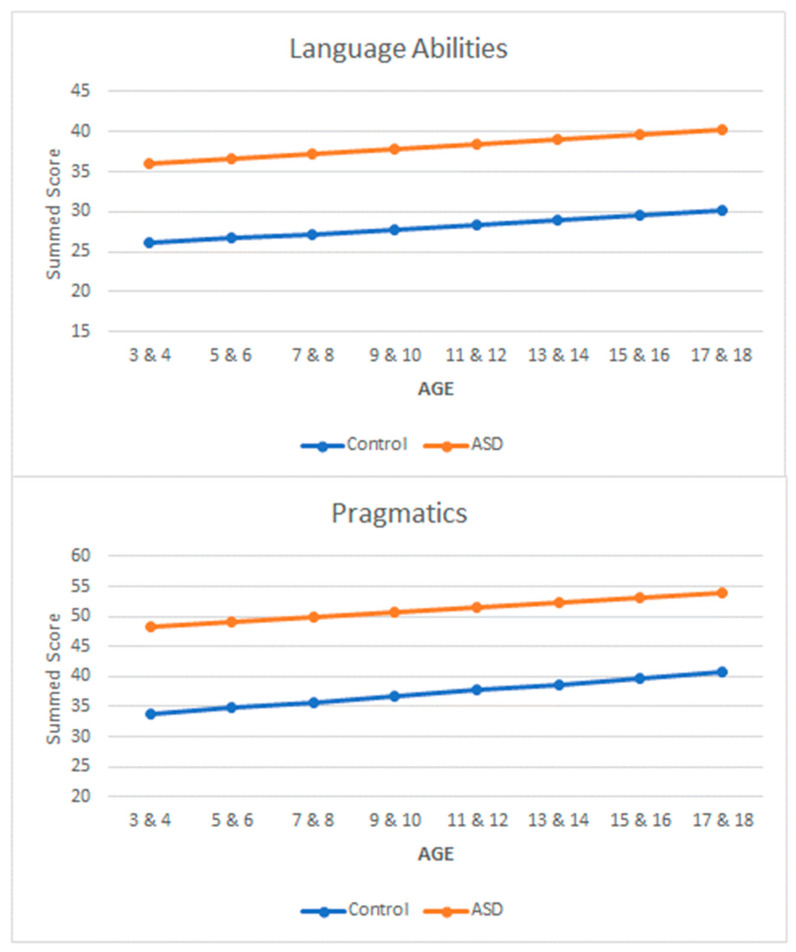
Interpretation plots based on the combined data from Experiments 1 and 2.

Turning to the issue of interpretation, we first had to address the fact that the final version of the questionnaire contained two fewer items than the version used in Experiment 2. Second, we pooled the data from both experiments to determine the expected intercepts and slopes. Essentially, we ran simple regressions, regressing age on language abilities and age on pragmatics. The results from our analyses are presented in Figure 5. As can be seen in Figure 5, we grouped age into two-year bins from 3 to 18 years of age (8 bins in total). Using these figures, a researcher or a clinician should be able to interpret the scores for a particular individual to determine whether they are at risk or not. Scores above the orange line would be a clear indicator of potential ASD symptomology and scores below the blue line would be an indicator of typical development. Individuals falling in the middle would be less clear, but useful information can be gained by proximity to either line. 

### 4.2. Strengths and Limitations 

The main strengths of this study are the large sample size for ASD participants, particularly for females in the ASD group(s), and the clear results relating to group differences. The effect sizes for language abilities were nearly identical for Experiment 1 and Experiment 2, and the pragmatics sub-scales showed even larger effect sizes and again, a high degree of similarity. There are a few limitations, and most are related to Experiment 2. The first is the missing age information in Experiment 2, and relatedly, the substantially younger age of control participants (for those reporting age). Moreover, those younger control participants were given quite low ratings for language abilities (i.e., parents rated the language of their child as being quite “good”). Thus, we ended up with overall lower means for Experiment 2, but particularly on language abilities. We took these factors into account when developing the interpretation figures (see Figure 5). Another potential limitation is concerned with demographic information. Participants were not asked whether their child had other potential neurodevelopmental conditions or disorders, just whether their child had no suspected or diagnosed ASD (Supplementary Materials—Sections B and D). We therefore cannot guarantee that individuals with ASD did not have comorbid disorders, nor that the control sample were all neurotypical. However, parents were asked whether their child had a speech and language disorder (24.7% answered yes for the ASD group and 12.9% answered yes for the control group).

There are also a few outstanding psychometric issues which need to be addressed in future studies. For reliability, the most obvious future direction is to assess test–retest reliability, but it may also be important to address other informant ratings (a type of interrater reliability), perhaps between parents or by gathering peer or teacher ratings of child behavior. For validity, the most important next step is to assess divergent validity. In our opinion, a key issue with respect to divergent validity would be a measure of anxiety, and thus, we would recommend a future study to examine the correlations between our questionnaire and anxiety measures. Psychometrically, we would expect anxiety to correlate with language abilities and pragmatics, but would not want those correlations to be as high as those observed for the AQ. It would also be possible to strengthen the convergent validity of our questionnaire by directly comparing it to one of the measures discussed in the introduction.

Finally, the discriminant analyses conducted in both experiments show the questionnaire to be diagnostically valid in terms of both sensitivity and specificity. The sensitivity of our questionnaire was 0.87 in Experiment 1 and 0.82 in Experiment 2. The specificity was 0.79 in Experiment 1 and 0.89 in Experiment 2. Thus, the questionnaire does a reasonably good job or better of identifying both individuals who actually have ASD and correctly classifying individuals who do not have ASD.

## 5. Conclusions

We believe that our questionnaire will be a useful tool because it can be used in both research and clinical settings and assesses (very broadly) the particular language issues experienced by children with ASD. Furthermore, because it relies on parental report, its administration takes approximately 10 min, which is extremely useful in both research and clinical settings. It could also be easily modified for teacher reports, and thus, could be also useful in an educational context, particularly for educators and/or educational psychologists, who would like to highlight language issues in a child for parents. We more than welcome further refinements of this questionnaire, and are happy to cooperate with any research groups who would like to use it or attempt to improve it. 

In summary, this study provided an initial assessment and validation of a new tool for measuring language abilities and pragmatic abilities in children. The instrument was shown, in two experiments, to have more than acceptable psychometric properties. Language is an important skill for success in every aspect of life, and individuals who cannot communicate effectively will experience distress, isolation, and difficulty functioning. Children, particularly those with ASD, need interventions that focus on language, but probably more importantly, on how to communicate effectively. Early intervention for children with ASD is crucial in predicting positive outcomes. The instrument reported in this study can be used in the screening process prior to these intervention strategies.

## Figures and Tables

**Table 1:** Age and gender for the ASD and control group.

	ASD (60)	Control (77)	Significance
Variable	Mean (SD)	Mean (SD)	
Age of child	11.50 (6.35)	10.55 (4.45)	*t*(134) = 1.02, *p* = 0.31
Gender of Child (% male)	46.7	74.0	*t*(135) = 3.39, *p* < 0.001

**Table 2:** Results of factor analyses.

Language Abilities		
	Eigenvalue	% of Variance
Factor 1	5.981	46.006
Factor 2	1.604	12.335
Factor 3	1.120	8.614
**Pragmatics**		
	Eigenvalue	% of Variance
Factor 1	9.558	50.308
Factor 2	1.458	7.673
Factor 3	1.040	5.475

**Table 3:** Factor Loadings: grey highlighting indicates significance.

Language Abilities.				Pragmatics			
	F1	F2	F3		F1	F2	F3
Item 1	0.579	−0.264	0.517	Item 15	0.553	0.495	−0.288
Item 2	0.624	−0.540	−0.138	Item 16	0.769	−0.386	−0.154
Item 3	0.690	−0.606	−0.041	Item 17	0.500	−0.099	0.525
Item 4	0.553	0.267	0.419	Item 18	0.754	0.112	−0.332
Item 5	0.630	−0.149	−0.591	Item 19 (R)	0.724	−0.283	−0.149
Item 6	0.794	−0.214	−0.121	Item 20	0.803	−0.030	0.137
Item 7				Item 21 (R)	0.864	0.108	0.025
Item 8 (R)	0.623	0.527	0.006	Item 22 (R)	0.559	−0.600	−0.107
Item 9 (R)	0.713	0.293	0.077	Item 23	0.710	0.111	−0.169
Item 10	0.860	−0.156	0.079	Item 24	0.801	−0.044	0.276
Item 11	0.615	0.199	0.058	Item 25	0.689	−0.044	−0.052
Item 12	0.742	0.242	−0.195	Item 26	0.708	−0.133	0.299
Item 13 (R)	0.575	0.449	−0.363	Item 27	0.498	0.480	0.253
Item 14	0.744	0.044	0.327	Item 28 (R)	0.628	0.135	−0.421
				Item 29 (R)	0.711	0.343	0.179
				Item 30 (R)	0.687	0.058	0.105
				Item 31 (R)	0.680	−0.034	−0.019
				Item 32	0.807	0.200	−0.091
				Item 33			
				Item 34	0.846	0.055	0.064

**Table 4:** Age and gender for the ASD and control group.

	ASD (70)	Control (27)	Significance
Variable	Mean (SD)	Mean (SD)	
Age of child ^1^	10.36 (5.67)	6.26 (3.96)	*t*(53) = -2.99, *p* = 0.004
Gender of Child (% male)	67.1	40.7	*t*(95) = −1.96, *p* < 0.052

*Note.*^1^ 48 parents did not report their child’s age.

**Table 5:** Results of factor analyses.

**Language Abilities**		
	Eigenvalue	% of Variance
Factor 1	5.989	46.069
Factor 2	1.587	12.206
**Pragmatics**		
	Eigenvalue	% of Variance
Factor 1	8.736	45.980
Factor 2	1.414	7.440
Factor 3	1.096	5.767

**Table 6:** Factor Loadings: grey highlighting indicates significance.

Language Abilities.			Pragmatics			
	F1	F2		F1	F2	F3
Item 1	0.626	−0.034	Item 14	0.657	0.246	0.308
Item 2	0.754	−0.546	Item 15	0.612	−0.288	−0.073
Item 3	0.761	−0.538	Item 16	0.423	−0.595	0.099
Item4	0.418	0.624	Item 17	0.745	0.030	0.073
Item 5	0.718	−0.169	Item 18 (R)	0.667	0.176	−0.251
Item 6	0.774	−0.002	Item 19	0.754	−0.210	0.026
Item 7 (R)	0.669	0.288	Item 20 (R)	0.755	−0.071	−0.072
Item 8 (R)	0.551	−0.108	Item 21 (R)	0.706	−0.099	−0.119
Item 9	0.827	−0.260	Item 22	0.326	0.275	0.770
Item 10	0.506	0.159	Item 23	0.773	−0.065	0.028
Item 11	0.717	0.437	Item 24	0.705	−0.415	0.363
Item 12 (R)	0.604	0.406	Item 25	0.716	0.028	−0.255
Item 13	0.769	0.193	Item 26 (R)	0.805	0.008	−0.076
			Item 27 (R)	0.698	0.257	0.093
			Item 28 (R)	0.536	0.561	0.024
			Item 29 (R)	0.641	0.297	−0.247
			Item 30	0.756	0.170	−0.135
			Item 31	0.747	−0.094	−0.109
			Item 32	0.661	−0.239	0.016

## Data Availability

The data presented in this study are available on request from the corresponding author.

## Supplementary Materials

The following supporting information can be downloaded at: https://www.mdpi.com/article/10.3390/brainsci13020196/s1, Section A: Table showing other available measures and their properties; Section B: Table A Demographic Information for Experiment 1; Section C: Omitted Items based on Factor Analysis Experiment 1, and Omitted Items based on Factor Analysis Experiment 2; Section D: Table B Demographic Information for Experiment 2; Section E: Language and Pragmatics Questionnaire (LAP version 1.1.0); Section F: Social Skills Questionnaire and Communication Questionnaire.
