# Supplementary material for: A Parental-Report Questionnaire for Language Abilities and Pragmatics in Children and Adolescents with Autism Spectrum Disorders
Source: Brain Sci. 2023 Jan 24;13(2):196. doi: 10.3390/brainsci13020196 (PMC9953798; doi:10.3390/brainsci13020196)
Supplement: Supplementary file 1 [file brainsci-13-00196-s001.zip › brainsci-2129284-supplementary.pdf]

Supplementary Materials

Section A:

The table below contains the major and minor measures used to assess language and pragmatics. The table contains much of the basic information about each test, and in our opinion, the main strengths and weaknesses. We have also included the new questionnaire that we present in this study in line 1 of the table. The associated references for the table are presented below.

Name of Test	Author	Pragmatic Abilities	Language Abilities	Age Range	Administrator	Cost	Length of Test	Strengths	Weaknesses
Language & Pragmatics Questionnaire	O'Shea et al. (2022)	Yes	Yes	3-18	Parent/Caregiver	Free	30 items.<br><br>10 minutes to administer	Quick to administer.<br><br>Does not require professional training.	Test-test reliability and divergent validity have not yet been assessed.
Social Communication Questionnaire [A]	Rutter et al. (2003)	Yes	No	>4 years (with a development al age greater than 2) [A]	Parent/Caregiver<br><br>Clinicians who give it are recommended to have Bachelors or Masters degree. [B]	~£200 [B]	40 items.<br><br>< 10 minutes to administer [A]	Quick to administer. [A]<br><br>Widely used in research.<br><br>Can be used on both verbal and non-verbal children.	Costly.<br><br>High number false positives.<br><br>Cut off for 'likely ASD' should be lowered for children < 5, suggesting it is not sensitive for younger children.
Broad Autism Phenotype Questionnaire [D]	Hurley et al. (2007).	Yes	No	>18 years old. [D]	Self-administered by adults. [D]	Free	36 items (12 subscales) [D]	Good internal consistency. [E]<br><br>Established as reliable tool for assessing Broad Autism Phenotypes. [E]	Is designed to identify traits of ASD in adults, rather than in children.<br><br>Developed to assess broad phenotypes of Autism in parents of AS children rather than identify ASD. [D]
Child's Communication Checklist-2 [F]	Bishop et al. (2003)	Yes	Yes	4-16 years. [F]	Parent/Caregiver [G]	~£200 [G]	70 items.<br><br>5-10 minutes [F]	Quick to administer. [F]<br><br>Broad research base, well validated and reliable, standardized scoring.	Costly. [G]<br><br>Can only be accessed by professionals with a Bachelors or Masters degree. [G]
Yale in vivo Pragmatic Protocol [H]	Schoen-Simmons et al. (2014)	Yes	No	9-17 years. [H]	Researcher or Examiner [H]	Free	30 minute conversation. [H]	Assesses different aspects of pragmatics (i.e. broad coverage of pragmatics).	Requires trained administrator, to identify key markers/cues.<br><br>Less established/weaker research base.
Pragmatic Rating Scale [I]	Landa (2013)	Yes	No	4-18 years. [I]	Researcher or Examiner [I]	Free	Semi-natural conversational interactions.	Assesses different aspects of pragmatics (i.e. broad coverage of pragmatics).	Requires two trained (blind) administrators for post-hoc coding.

									Time consuming scoring.<br><br>Less established/weaker research base.
Pragmatics Profile of Everyday Communication Skills. [J,K]	Dewart & Summers (1996); Almehmadi et al. (2020)	Yes	No	9 months – 10 years. [K]	Original format Researcher / Examiner interview parent/caregiver. Modified to questionnaire. [K]	Free	22 Questions.<br><br>10-15 minutes. [K]	Quick to administer. [K]<br><br>Broad coverage of communication skills.	Modifications not as well validated as original interview schedule. [K]<br><br>Less established/weaker research base.
Language Use Inventory [L]	O'Neill (2007)	Yes	No	1-4 years. [L]	Caregiver. [L]	~£200 [M]	180 Questions.<br><br>20-30 minutes. [M]	Broad research base, well validated and reliable, standardized scoring. [M]	Costly.<br><br>Can only be accessed by professionals with a Bachelors or Masters degree.<br><br>Small age range. [L]
Test of Pragmatic Language [N]	Phelps Terasaki, and Phelps-Gunn (2007)	Yes	No	6-18;11 years.[N]	Researcher or Therapist. The administrator should have a Doctorate or licencing to administer the test. [O]	~£300 [O]	40-60 minutes [O]	Norm referenced test. [O]<br><br>Broad research base, well validated and reliable, standardized scoring.	Costly.<br><br>Must be administered by a trained professional. [O]<br><br>Takes a relatively long time to administer. [O]
Pragmatic Language Skills Inventory [P]	Gilliam and Miller (2006)	Yes	No	5-12 years. [P]	Teacher, caregiver or clinician. However, test purchaser must have a Bachelors degree in psychology or related field. [Q]	~£110 [Q]	45 items.<br><br>5-10 minutes. [Q]	Quick to administer. [Q]<br><br>Norm referenced. [P]<br><br>Broad research base, well validated and reliable, standardized scoring	Costly. [Q]<br><br>Does not assess language/only focuses on interactions. [P]<br><br>Can only be used on a relatively small age range of children. [P]

[A] Rutter, M., Bailey, A., & Lord, C. (2003). *The social communication questionnaire*. Los Angeles, CA: Western Psychological Services.
[B] WPS. (2023). (SCQ) Social Communication Questionnaire. (SCQ) Social Communication Questionnaire (wpspublish.com)
[C] Children's Hospital of Philadelphia. (2020). Social Communication Questionnaire (SCQ). Social Communication Questionnaire (SCQ) | CHOP Research Institute.
[D] Hurley , R. S., Losh, M., Parlier, M., Reznick, J. S., & Piven, J. (2007). The broad autism phenotype questionnaire. *Journal of autism and developmental disorders*, *37*(9), 1679-1690.
[E] Sasson, N. J., Lam, K. S. L., Childress, D., Parlier, M., Daniels, J. l., & Piven, J. (2013). The Broad Autism Phenotype Questionnaire: Prevalence and Diagnostic Classification. *Autism Research, 6*(2), 134-143.
[F] Bishop, D. V. M. (2003). The children's communication checklist. London: Harcourt Assessment.
[G] Pearsons. (2023). Children's Communication Checklist. https://www.pearsonclinical.co.uk/store/ukassessments/en/c/Children%27s-Communication-Checklist/p/P100009204.html
[H] Schoen-Simmons, E., Paul, R., & Volkmar, F. (2014). Assessing pragmatic language in autism spectrum disorder: the Yale in vivo Pragmatic Protocol. *Journal of speech, language, and hearing research : JSLHR, 57*(6), 2162–2173. https://doi.org/10.1044/2014_JSLHR-L-14-0040
[I] Landa, R. (2013). Pragmatic rating Scale for school aged children coding sheet, revised. Baltimore, MD: Kennedy Kreiger Institute.
[J] Dewart, H., & Summers, S. (1996). The pragmatics profile of everyday communication skills in adults. Windsor: NFER Nelson.
[K] Almehmadi, W., Tenbrink, T., & Sanoudaki, E. (2020). Pragmatic and Conversational Features of Arabic-Speaking Adolescents With Autism Spectrum Disorder: Examining Performance and Caregivers' Perceptions. *Journal of Speech, Language, and Hearing Research, 63*, 2308-2321.
[L] O'Neill, D.K. (2007). The language use inventory for young children: A parent-report measure of pragmatic language development for 18- to 47-month old children. *Journal of Speech, Language, and Hearing Research*. https://pubs.asha.org/doi/10.1044/1092-4388%282007/017%29
[M] Language Use Inventory. (2023). *About the Language Use Inventory.* https://languageuseinventory.com/AboutTheLUI
[N] Phelps Terasaki, D., & Phelps-Gunn, T. (2007). Test of Pragmatic Language, Second Edition. Austin, TX: Pro-Ed.
[O] WPS. (2023). *(TOPL-2) Test of Pragmatic Language, Second Edition*. https://www.wpspublish.com/topl-2-test-of-pragmatic-language-second-edition
[P] Gilliam, J. E., & Miller, L. (2006). Pragmatic Language Skills Inventory. Austin, TX: Pro-Ed.
[Q] pro.ed. (2023). *PSLI: pragmatic Language Skills Inventory.* https://www.proedinc.com/Products/11365/plsi-pragmatic-language-skills-inventory.aspx

Section B:

Table A

Demographic Information for Experiment 1.

Variable	Controls (60)	ASD (77)
Gender of parent		
Male	1(1.7)	7(9.1)
Female	59 (98.3)	70(90.9)
Number of children	2.17 (.72)	2.36 (.83)
Age of parent		
18-24	2 (3.3)	1 (1.3)
25-30	0 (0)	3 (3.9)
31-40	15 (25)	28 (36.4)
41-50	38 (63.3)	36 (46.8)
51-50	4 (6.7)	7 (9.1)
61 or over	1 (1.7)	2 (2.6)
Parent's income		
Low	17 (28.3)	29 (37.7)
Low-average	22 (36.7)	32 (41.6)
High-Average	17 (28.3)	12 (15.6)
High	2 (3.3)	4 (5.2)
Household income		
Low	4 (6.7)	11 (14.3)
Low-average	12 (20)	36 (46.8)
High-average	35 (58.3)	24 (31.2)
High	8 (13.3)	5 (6.5)
Parent Education		
Less than school	1 (1.7)	2 (2.6)
GCSE's/A Levels	18 (30)	22 (28.6)
Certificate	4 (6.7)	9 (11.7)
Diploma	6 (10)	16 (20.8)
Bachelor's degree	21 (35)	21 (27.3)
Master's degree	9 (15)	6 (7.8)
Doctoral degree	0 (0)	1 (1.3)

Section C:

Omitted Items based on Factor Analysis Experiment 1.

7. My child has developed a strong vocabulary in an area of interest very quickly
33. My child makes up their own words

Omitted Items based on Factor Analysis Experiment 2.

4. My child does not respond to their own name
22. My child does not use communicative or symbolic gestures

Section D:

Table B

Demographic Information for Experiment 2.

		Suspected or Diagnosed ASD		No Suspected or Diagnosed ASD	
		N	*Percentage*	*N*	*Percentage*
Child Gender	Male	50	68.5%	12	38.7%
	Female	21	28.8%	19	61.3%
	Other	2	2.7%	0	0%
Child Ethnicity	Mixed/Multiple Ethnic Groups	5	6.8%	4	12.9%
	White	67	91.8%	27	87.1%
	Asian	0	-	-	-
	Black/African/Caribbean	1	1.4%	-	-
Relation to Child	Mother	72	98.6%	28	90.3%
	Father	0		2	6.5%
	Other	1	1.4%	1	3.2%
Parental Qualification Level	GCSEs/O-levels	12	16.4%	5	16.1%
	A-levels	11	15.1%	4	12.9%
	Undergraduate Degree	27	37.0%	8	25.8%
	Postgraduate Degree	20	27.4%	10	32.3%
	PHD	3	4.1%	4	12.9%
Household Income	£10,000-£19,999	9	12.3%	7	22.6%
	£20,000-£29,999	12	16.4%	3	6.5%
	£30,000-£39,999	9	12.3%	5	16.1%
	£40,000-£49,999	9	12.3%	0	0%
	£50,000-£99,999	21	28.8%	14	45.2%
	£100,000+	11	15.1%	2	6.5%
Speech and Language Disorder	Yes	18	24.7%	4	12.9%
	No	55	75.3%	27	87.1%
SLT Input	Yes	44	60.3%	7	22.6%
	No	29	39.7%	24	77.4%
Hours of SLT Input	Less than 5 hours	11	15.1%	2	6.5%
	6-10 hours	10	13.7%	1	3.2%
	21-30	8	11.0%	2	6.5%
	31+ hours	14	19.2%	2	6.5%
Verbal Older Siblings	Yes	35	47.9%	17	54.8%
	No	38	52.1%	14	45.2%

Number of Older Siblings	1	21	28.8%	11	35.5%
	2	9	12.3%	6	19.4%
	3	2	2.7%	-	-
	4+	2	2.7%	-	-
	N/A	1	1.4%	-	-
Read to Child	Yes	73	100%	31	100%
	No	0	-	0	-
Frequency of Reading	Everyday	54	74%	24	77.4%
	2-6 times a week	17	23.3%	6	19.4%
	Less than twice a week	2	2.7%	1	3.2%

Section E:

Language and Pragmatics Questionnaire (LAP version 1.1.0)

Sex of your child: Male/Female

Age of your child:

Age of diagnosis:

How to fill out the questionnaire

Below is a list of statements. Please read each statement very carefully and rate how strongly you agree or disagree with it by circling your answer.

Language ability:

1) My child does not speak or uses very limited speech
2) My child's first words were delayed
3) My child was a late speaker compared to children of the same age
4) At some point, my child has been in the low ability reading group at school
5) The teacher has expressed concern about my child's language abilities
6) My child is responsive to the speech of others
7) My child's vocabulary is similar to his/her peers
8) As a parent, I have felt concerned about my child's language development
9) My child has experienced the loss of words they were previously able to say
10) Understanding simple statements and answering questions is hard for my child
11) My child is able to follow directions and find objects that are named
12) Verbal communication is difficult for my child

Pragmatics:

13) Non-verbal communication is difficult for my child
14) My child says things with little or no content or information.
15) I have heard my child imitating learned scripts, such as those heard during television adverts
16) It is difficult for my child to communicate their needs and desires
17) My child is able to stay on topic during conversations and storytelling
18) My child uses odd phrases and choices of words

19) My child is able to change their language appropriately according to the needs of a listener or situation

20) My child is able to initiate and hold fluent conversations

21) My child demonstrates one-sided interactions

22) My child repeats words or phrases without communicative intent

23) My child speaks in an abnormal tone of voice, or with an off rhythm or pitch

24) It is difficult for my child to understand/monitor the emotions of others

25) My child is able to produce emotional language

26) It is not difficult for my child to share their emotions with others

27) My child is able to make eye contact with people and objects

28) Peer interaction is easy for my child

29) My child takes what is said too literally, often missing the humour, irony, and sarcasm

30) My child uses unusual or inappropriate body language, gestures, and facial expressions

---

Scoring:

4 = Strongly agree 3= Agree 2= Disagree 1= Strongly disagree

Reverse scoring for items in blue font

Section F:

Social Skills Questionnaire

1. My child prefers to do things with others rather than on their own.
2. My child finds social situations easy.
3. My child would rather go to a library than a birthday party.
4. My child is drawn more strongly to people than things.
5. My child finds it hard to make new friends.
6. My child finds it easy to work out what someone is thinking just by looking at their face.
7. My child enjoys social situations.
8. My child finds it difficult to work out people's intentions
9. My child enjoys meeting new people.
10. My child is a good is good at taking care not to hurt other people's feelings diplomat

Communication Questionnaire

1. Other people frequently tell my child what they've said is impolite.
2. My child enjoys social chit-chat.
3. When my child talks, it isn't always easy for others to get a word in edgeways.
4. My child struggles to keep a conversation flowing with their peers.
5. My child finds it easy to 'read between the lines' when someone is talking to them.
6. My child knows how to tell if someone listening to them is getting bored.
7. When my child talks on the phone, they are not sure when it is their turn to speak.
8. My child is often the last to understand the point of a joke.
9. My child is good at social chit-chat.
10. People often tell my child that they are going on about the same thing.
